# Effects of Dl-3-n-butylphthalide on neurological function, hemodynamics and Hcy concentration in cerebral hemorrhage: a systematic review and meta-analysis

**DOI:** 10.3389/fphar.2024.1360932

**Published:** 2024-05-30

**Authors:** Yingqi Ma, Chenchen Guo, Yiguo Wang, Xinxin Liu

**Affiliations:** ^1^ First School of Clinical Medicine, Shandong University of Traditional Chinese Medicine, Jinan, Shandong, China; ^2^ Neck-Shoulder and Lumbocrural Pain Hospital of Shandong First Medical University, Shandong First Medical University and Shandong Academy of Medical Sciences, Jinan, China; ^3^ Experimental Research Center, China Academy of Chinese Medical Sciences, Beijing, China; ^4^ Centre for Evidence-Based Chinese Medicine, Beijing University of Chinese Medicine, Beijing, China

**Keywords:** Dl-3-n-butylphthalide, cerebral hemorrhage, neurological function, meta-analysis, systematic review

## Abstract

**Background:**

Dl-3-n-Butylphthalide (NBP) has emerged as a potential therapeutic agent for cerebral hemorrhage, despite not being included in current guideline recommendations. Investigating the underlying physiological and pathological mechanisms of Dl-3-n-Butylphthalide in cerebral hemorrhage treatment remains a critical area of research.

**Objective:**

This review aims to evaluate the efficacy of Dl-3-n-Butylphthalide in cerebral hemorrhage treatment and elucidate its potential biological mechanisms, thereby providing evidence to support treatment optimization.

**Methods:**

A comprehensive search of seven electronic databases (PubMed, Web of Science, Embase, Cochrane Library, China National Knowledge Infrastructure, VIP, and Wanfang Database) was conducted for studies published up to September 2023. Screening and data extraction were performed by a team of researchers. The Cochrane collaboration tool was utilized for risk bias assessment, and Revman 5.3 along with Stata 17.0 were employed for statistical analysis.

**Outcomes:**

We searched 254 literature, and 19 were included in this meta-analysis. The results showed that Dl-3-n-Butylphthalide improved the clinical efficacy rate (RR = 1.25, 95% CI 1.19–1.31; *p* = 0.00), quality of life (MD = 13.93, 95% CI: 11.88–15.98; *p* = 0.000), increased cerebral blood flow and velocity, reduced cerebral edema volume, Hcy concentration, and did not have obvious adverse reactions (RR = 0.68, 95% CI: 0.39–1.18; *p* = 0.10).

**Conclusion:**

This meta-analysis is the first to demonstrate the potential of Dl-3-n-Butylphthalide in treating cerebral hemorrhage. It suggests that Dl-3-n-Butylphthalide may alleviate clinical symptoms by modulating neurological function and improving hemodynamics. Our findings provide robust evidence for incorporating Dl-3-n-Butylphthalide into cerebral hemorrhage treatment strategies, potentially guiding future clinical practice and research.

**Systematic Review Registration: https://www.crd.york.ac.uk/PROSPERO/ display_record.php?RecordID=355114, Identifier CRD42022355114.**

## 1 Introduction

Cerebral hemorrhage, a severe form of stroke, is categorized into non-traumatic and traumatic types. It typically manifests with sudden local neurological dysfunction and potential elevations in intracranial pressure. Clinical symptoms often include nausea, vomiting, headache, increased blood pressure, and cognitive impairment (Hwang et al., 2023; Romero and Rojas-Serrano, 2023). Non-traumatic intracerebral hemorrhage (ICH) primarily results from ruptured arterial vessels, frequently occurring in the basal ganglia or thalamic regions. The predominant cause is cerebral small vessel diseases (CSVD), affecting small arteries. Conditions such as cerebral amyloid angiopathy (CAA) and hypertensive arteriosclerosis are common examples (DeSimone et al., 2017; Gil-Garcia et al., 2022). Approximately 20% of ICH cases arise from other etiologies, including arteriovenous malformations, spongiomas, or fistulas (Sallinen et al., 2020). ICH constitutes about 20% of all stroke incidents and remains a significant cause of morbidity and mortality globally, with mortality rates reaching up to 50% (Poon et al., 2014; Hemphill et al., 2015).The incidence of ICH varies regionally, with the Global Burden of Disease Study reporting that 80% of global brain hemorrhages occur in low-and middle-income countries (Krishnamurthi et al., 2014). By 2050, the incidence is projected to increase by 35.2%. The aging population trend suggests that the proportion of cerebral hemorrhage patients over 80 years will rise by 2.5 times (Steiner et al., 2016). Traumatic intracerebral hemorrhage results from external head impacts, often leading to contusions in the frontal and temporal lobes. Concurrently, patients may exhibit neuroimaging signs of traumatic subarachnoid hemorrhage, epidural hematoma, subdural hematoma, or skull fractures (Cepeda et al., 2015).

Early hematoma expansion is a prevalent and grave complication in cerebral hemorrhage patients, often indicating a dismal prognosis (Chen et al., 2015). To mitigate hematoma growth, clinicians administer hemostatic agents such as tranexamic acid, recombinant activated factor VII (rFVIIa), and prothrombin complex concentrate (PCC). However, these treatments minimally impact the improvement of clinical symptoms and lack sufficient evidence to demonstrate effects on functional outcomes or mortality reduction (Steiner and Bsel, 2010; Garg and Biller, 2019). The “mass effect,” inflammatory responses, and iron-induced secondary damage (Jiang et al., 2020) necessitate medical interventions targeting secondary injury. These include anti-inflammatory agents (Ironside et al., 2021) and iron chelating agents (Ramadhan et al., 2023). Surgical evacuation of the hematoma represents a therapeutic approach for cerebral hemorrhage. Yet, current evidence scarcely supports that surgery or pharmacological treatment substantially decreases mortality or morbidity associated with cerebral hemorrhage (Wilkinson et al., 2018a; Wilkinson et al., 2018b; de Oliveira Manoel, 2020). Therefore, exploring novel methods to enhance neuroinflammation management and neural function recovery post-cerebral hemorrhage is imperative.

In mitigating adverse neurological outcomes and mortality among cerebral hemorrhage patients, prompt diagnosis and swift implementation of suitable interventions are vital (Rabinstein, 2017). Despite extensive clinical research in recent years investigating optimal treatment strategies for cerebral hemorrhage, including Western medicine and surgical approaches, definitive evidence-based medical guidance for managing this condition remains elusive. Enhancing patient survival and quality of life continues to present significant challenges, underscoring the urgent necessity for novel therapeutic agents to augment current clinical outcomes (Al-Kawaz et al., 2020; Gil-Garcia et al., 2022; Magid-Bernstein et al., 2022; Sheth, 2023).

NBP, commonly known as butylphthalide, is an organic compound derived from celery oil. In 2002, the China Food and Drug Administration (CFDA) approved NBP for the treatment of cerebral infarction (Wang et al., 2018). Numerous studies have identified NBP as a promising neuroprotective agent, effective in treating heart failure, ischemic stroke, Parkinsonism syndrome, Alzheimer’s disease, and other neurologic disorders associated with cognitive dysfunction (Qiu et al., 2018; Wang et al., 2018; Gao et al., 2019; Li et al., 2022; Hu et al., 2023). Previous meta-analyses highlight NBP’s role in cerebrovascular disease treatment, including reducing inflammation, mitigating oxidative stress, and enhancing vascular endothelial function (Liu et al., 2023). Its neuroprotective effects in cerebrovascular diseases, corroborated by extensive animal studies, are attributed to a range of biomolecular mechanisms (Cheng et al., 2019; Liu et al., 2021; Chen X. et al., 2022a; Gao et al., 2022).

Tu et al. (2020) discovered that NBP may mitigate neurological impairment following localized cerebral hemorrhage by fostering neovascularization near the hemorrhage site. NBP can diminish brain cell apoptosis, safeguard neuronal cells, and repair neurological function, primarily through the upregulation of UBIAD1 expression. However, there has been limited investigation into the therapeutic potential of this compound in experimental models of hemorrhagic stroke (Pi et al., 2021).

To our knowledge, no meta-analysis has specifically addressed the efficacy of NBP in treating cerebral hemorrhage. This study serves as a reference for clinicians considering NBP as a therapeutic option for this condition.

## 2 Methods

This study has been registered on the PROSPERO platform (Registration No. CRD42022355114, https://www.crd.york.ac.uk/PROSPERO/ display_record.php?RecordID=355114).

### 2.1 Eligibility criteria

#### 2.1.1 Inclusion criteria and exclusion criteria

(1) Patients: All included patients must conform to the established Western medical diagnostic criteria for cerebral hemorrhage; Age ≥18 years old. Meanwhile, patients with severe cardiac, hepatic, or renal insufficiency; patients with malignant tumors or severe organic lesions; patients with severe neurological dysfunction or a history of psychiatric disorders; patients with autoimmune diseases, diabetes mellitus, and acute infectious diseases; and patients with allergy to NBP were excluded.
(2) Interventions: The treatment group was given NBP injection (25 mL/bid) or NBP soft capsule (0.2 g/tid) on top of the control group.
(3) Comparisons: The control group was given routine treatment (maintaining stable vital signs, mannitol dehydration, maintaining water electrolytes, acid-base balance, nutritional support, and nutritional brain cells).
(4) Outcome measures: ①Clinical efficiency; ②Incidence of adverse reactions; ③National Institutes of Health Stroke Scale (NIHSS); ④Activity of Daily Living Scale (ADLS); ⑤Mean cerebral blood flow (MCBF); ⑥Mean cerebral blood flow velocity (MBFV); ⑦Cerebral edema volume; ⑧Hcy concentration; ⑨SP concentration.
(5) Studies: Randomized Controlled Trials (RCTs). The following were excluded: case reports, conference papers and animal studies, etc.;

### 2.2 Literature search

Seven electronic databases including PubMed, Web of Science, Embase, Cochrane Library, CNKI, VIP, and Wanfang Database were searched by computer from the establishment of the database to September 2023. Pubmed search strategy: (((((((Cerebral Hemorrhage [MeSH Terms])) OR (Cerebral Hemorrhage [Title/Abstract])) OR (Cerebrum Hemorrhage [Title/Abstract])) OR (Cerebral Parenchymal Hemorrhage [Title/Abstract])) OR (Intracerebral Hemorrhage [Title/Abstract])) OR (Cerebral Brain Hemorrhage [Title/Abstract])) AND ((((((((3-n-butylphthalide [MeSH Terms])) OR (3-n-butylphthalide [Title/Abstract])) OR (DL-3-n-butylphthalide [Title/Abstract])) OR (l-NBP cpd [Title/Abstract])) OR (N-butylphthalide [Title/Abstract])) OR (butylphthalide [Title/Abstract])) OR ((S)-(−)-3-butylphthalide [Title/Abstract])).

### 2.3 Literature screening and data extraction

We imported the retrieved literature into EndNote 20.1 and excluded those not meeting the eligibility criteria. Two researchers, C-C G and Y-G W, independently reviewed the titles and abstracts to identify studies suitable for analysis. Concurrently, all full texts meeting the inclusion criteria were downloaded and independently assessed. Researchers C-C G and Y-Q M utilized Excel 2021 to collate and organize the baseline characteristics of the selected studies, which included the first author, publication year, sample size, region, study design, participants’ gender and age, blood loss, intervention types, treatment duration, and outcomes. The results were cross-verified, and any discrepancies were resolved through discussion between the parties involved. If necessary, a third researcher (Y-Q M) was available to assist in resolving any disagreements.

### 2.4 Quality evaluation

Two researchers, Y-G W and C-C G, evaluated the quality of the included studies utilizing the Revman5.3 Risk of Bias assessment tool. The assessment criteria encompassed random sequence generation, allocation concealment, blinding (including implementer and participant blinding, as well as outcome evaluator blinding), incomplete outcome data, selective reporting, and other potential sources of bias. Based on this quality assessment, the risk levels were categorized as “low risk,” “high risk,” or “unclear risk.” In cases of disagreement, the two researchers engaged in discussions, and a third researcher, X-X L, intervened as necessary to make a final determination.

### 2.5 Statistical analysis

The meta-analysis was conducted using Stata 17.0 software. Overall parameter estimates were presented as 95% confidence intervals (95% CI), with *p*-values less than 0.05 indicating statistical significance. (1) The I^2^ test was employed to assess heterogeneity among studies. A fixed-effect model was applied when heterogeneity was low (*p* > 0.1, I^2^ < 50%). In contrast, a random-effects model was chosen in cases of high heterogeneity (*p* < 0.1, I^2^ > 50%). (2) The effect size for binary variables was measured using Relative Risk (RR), and Mean Difference (MD) for continuous variables. Standardized Mean Difference (SMD) was utilized when the included studies employed different measurement instruments, whereas Weighted Mean Difference (WMD) was used otherwise.

### 2.6 Sensitivity analysis

To assess the stability and reliability of the outcome indicators, sensitivity analysis was conducted. In instances of high heterogeneity, a Galbraith plot was utilized to identify the sources of significant heterogeneity in each study.

### 2.7 Meta-regression analysis

Meta-regression analysis was conducted on variables such as age, sex, and route of administration in studies comprising more than ten reports, to determine their correlation with the effect size.

### 2.8 Publication bias

If the funnel plot exhibits asymmetry or the Egger test indicates publication bias (*p* < 0.05), it becomes necessary to assess the stability and reliability of the outcome indicators. In such cases, the “Trim and Fill” method is employed to estimate the combined effect size while adjusting for publication bias.

## 3 Results

### 3.1 Results of literature search

**FIGURE 1:**
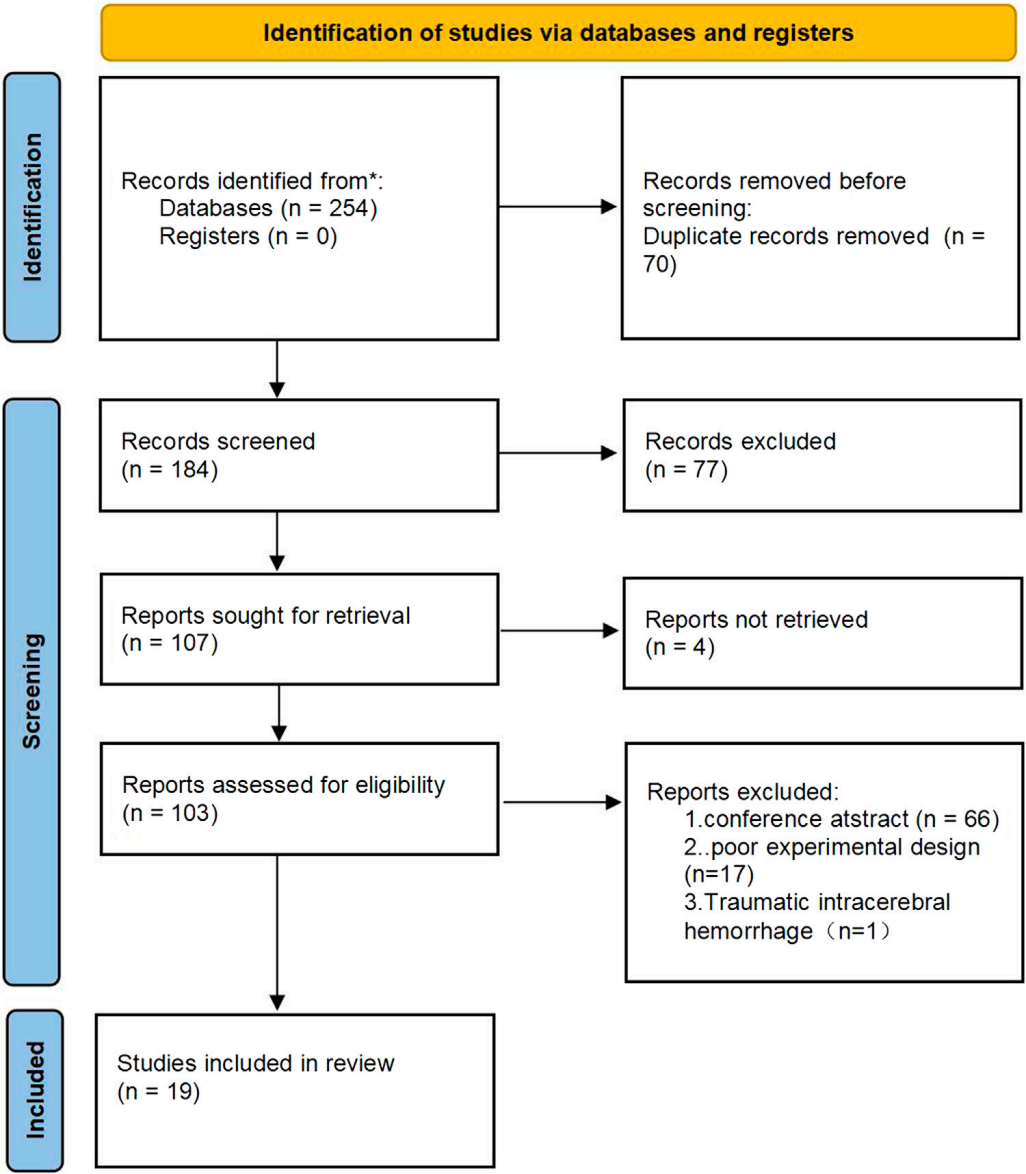
Flow chart of literature screening.

A comprehensive computer search yielded 254 studies. After the exclusion of 70 duplicates, the titles, abstracts, and full texts of the remaining 184 articles were meticulously reviewed. Studies that failed to meet the inclusion criteria were subsequently excluded. Ultimately, 19 studies qualified for inclusion. The detailed retrieval process is illustrated in Figure 1.

### 3.2 Baseline characteristics

The selected studies, published between 2017 and 2023, exclusively involved Chinese patients. This meta-analysis incorporated 19 randomized controlled trials (Wang Y. et al., 2021a; Qian, 2021; Liu et al., 2018; Pang et al., 2010; hongli, 2019; Han et al., 2021; yan et al., 2021; Shi and Chen, 2022; Qian et al., 2017; Chen et al., 2018; Huang et al., 2022; yang et al., 2020; Lin, 2021; Guo and Liao, 2022; fang, 2017; Jia et al., 2017; fang and yixin, 2021; Ji and Zhou, 2017; Chen et al., 2023), with 17 published in Chinese and two in English. The studies collectively enrolled 1959 patients diagnosed with cerebral hemorrhage, aged between 46 and 73 years. The sample sizes varied, ranging from 60 (Pang et al., 2010; Guo and Liao, 2022; fang, 2017) to 192 (Chen et al., 2018). Among these, 1742 patients were diagnosed with ICH (Qian, 2021; Liu et al., 2018; Pang et al., 2010; hongli, 2019; Han et al., 2021; yan et al., 2021; Shi and Chen, 2022; Qian et al., 2017; Chen et al., 2018; Huang et al., 2022; yang et al., 2020; Lin, 2021; Guo and Liao, 2022; fang, 2017; Jia et al., 2017; fang and yixin, 2021; Ji and Zhou, 2017), while 217 patients had traumatic intracerebral hemorrhage (Wang Y. et al., 2021a; Chen et al., 2023). The intervention in all studies was a combination of NBP and conventional therapy. Table 1 presents the baseline characteristics of these studies.

**TABLE 1:** Baseline characteristics of the included studies.

Study	disease	Region	study type	Sample Size	Gender(%F)	Age[mean(SD)]	Intracranial bleeding (ml)	Course of hypertension (yd)	Onset time(h)	Intervention	Dose/Duration(days)	follow-up time(days)	Outcomes
Wang YH, et al., 2021 (Wang et al., 2021a)	HICH	Zhejiang	RCT	115	40.87	T:60.21 ± 10.61 C:61.41 ± 9.85	NA	T: 6.95 ± 2.17 C:6.79 ± 1.86	T:7.32 ± 1.95 C:7.65 ± 2.03	T:NBP injection + nimodipine; C: nimodipine	25 ml/bid 14d	NA	①②③
Zhang Q 2021 (Qian, 2021)	ICH	Henan	RCT	88	35.23	T:56.37 ± 3.07 C:54.12 ± 2.34	T:30.06 ± 5.14 C:29.34 ± 4.35	NA	NA	T:NBP soft capsule + Nimodipine; C: Nimodipine	0.2 g/tid 70d	NA	④⑤⑥
Liu CY, et al., 2018 (Liu et al., 2018)	aSAH	Hubei	RCT	63	58.73	T:46.9 ± 10.8 C:50.1 ± 10.8	NA	NA	NA	T:NBP injection + CT; C:CT	25 ml/bid 7d	28	①
Pang QJ, et al., 2010 (Pang et al., 2010)	HICH	Hebei	RCT	60	41.67	NA	NA	NA	NA	T:NBP soft capsule + CT; C: CT	0.2 g/tid 20d	21	①④
Xu HL 2019 (hongli, 2019)	SCH	Henan	RCT	98	39.79	T:58∼79 C:57∼80	NA	NA	T:2∼12 C:2∼16	T:NBP injection + naloxone; C: naloxone	25 ml/bid 14d	NA	①
Han HB et al., 2021 (Han et al., 2021)	SCH	Henan	RCT	110	63.64	T:67.55 ± 3.56 C:68.43 ± 3.67	T:41.43 ± 4.82 C:43.64 ± 4.75	NA	NA	T:NBP injection + HOT; C: HOT	25 ml/bid 14d	NA	①②③④⑤⑥⑨
Liu Y_a_, et al., 2021 (yan et al., 2021)	ICH	Neimenggu	RCT	140	42.14	T:68.9 ± 1.2 C:68.7 ± 1.2	NA	NA	NA	T:NBP injection + oxiracetam; C: oxiracetam	25 ml/bid 21d	NA	①④
Shi L, et al., 2022 (Shi and Chen, 2022)	SCH	Shandong	RCT	84	46.43	T:73.51 ± 9.14 C:73.89 ± 9.35	T:18.50 ± 2.65 C:18.21 ± 2.77	T:8.65 ± 3.24 C:8.89 ± 3.16	NA	T:NBP injection + oxiracetam; C: oxiracetam	25 ml/bid 14d	NA	①②③④
Qian QQ, et al., 2017 (Qian et al., 2017)	SCH	Hebei	RCT	130	42.31	T:66.5 ± 3.2 C:66.8 ± 3.9	NA	NA	T:7.7 ± 3.0 C:6.8 ± 3.1	T:NBP injection + naloxone; C: naloxone	25 ml/bid 14d	NA	①③⑧
Chen FY, et al., 2018 (Chen et al., 2018)	ICH	Zhejiang	RCT	192	41.15	T:52.11 ± 9.82 C:52.80 ± 10.51	NA	NA	T:21.35 ± 10.52 C:22.12 ± 11.24	T:NBP injection + CT; C: CT	25 ml/bid 14d	NA	①③
Huang ZY, et al., 2022 (Huang et al., 2022)	ICH&PI	Guangdong	RCT	124	37.09	T:56.54 ± 6.28 C:56.47 ± 6.32	T:35.84 ± 3.43 C:35.78 ± 3.41	NA	T:21.39 ± 3.34 C:21.35 ± 3.28	T:NBP injection +CT; C: CT	25 ml/bid 14d	NA	①②③
Liu Y_b_, et al., 2020 (yang et al., 2020)	HICH	Anhui	RCT	100	49	T:76 ± 3 C:77 ± 3	T:25 ± 4 C:26 ± 3	NA	NA	T:NBP soft capsule + edaravone; C: edaravone	0.2 g/tid 28d	NA	①⑤⑥
Lin Q 2021 (Lin, 2021)	HICH	Zhejiang	RCT	180	45	T:72.25 ± 2.22 C:71.19 ± 2.36	NA	NA	NA	T:NBP injection + oxiracetam; C: oxiracetam	25 ml/bid 14d	NA	①②
Guo WC, et al., 2022 (Guo and Liao, 2022)	HICH	Guangdong	RCT	60	31.67	T:56.39 ± 6.91 C:53.61 ± 6.57	NA	NA	NA	T:NBP injection + MICLD; C:MICLD	25 ml/bid 14d	NA	①③⑦⑧⑨
Jian F 2017 (fang, 2017)	HICH	Chongqing	RCT	60	41.67	35∼72	NA	NA	NA	T:NBP soft capsule + CT; C: CT	0.2 g/tid 28d	NA	①
Jia YH, et al., 2017 (Jia et al., 2017)	HICH	Shanxi	RCT	80	45	T:63.7 ± 8.4 C:64.2 ± 7.5	NA	NA	NA	T:NBP injection + CT; C: CT	25 ml/bid 14d	28	①⑦⑧⑨
Huang F, et al., 2021 (fang and yixin, 2021)	HICH	Hunan	RCT	110	39.09	T:72.74 ± 8.15 C:71.34 ± 7.43	T:38.13 ± 6.75 C:36.34 ± 5.43	T:7.34 ± 3.43 C:7.84 ± 4.26	NA	T:NBP injection + oxiracetam; C: oxiracetam	25 ml/bid 14d	NA	①③④⑦⑧⑨
Ji XT, et al., 2017 (Ji and Zhou, 2017)	HICH	Hainan	RCT	63	42.86	T:59.92 ± 5.87 C:61.03 ± 5.72	NA	NA	NA	T:NBP injection + Xingnaojing injection; C: Xingnaojing injection	25 ml/bid 14d	NA	①③⑥⑦
Chen H, et al., 2023 (Chen et al., 2023)	SAH	Hunan	RCT	102	43.14	T:62.52 ± 12.38 C:63.24 ± 12.77	NA	NA	NA	T:NBP injection + nimodipine; C: nimodipine	25 ml/bid 10d	NA	①⑥

Note: ICH: intracerebral hemorrhage; HICH: Hypertensive intracerebral hemorrhage; aSAH: Aneurysmal subarachnoid hemorrhage; SCH: senile cerebral haemorrhage; ICH&PI; Cerebral hemorrhage complicated with pulmonary infection; SAH: subarachnoid hemorrhage; CT: conventional therapy; HOT: Hyperbaric Oxygen Therapy; MICLD: minimally invasive catheter suction liquefaction drainage; RCT: Randomized controlled trial; NA: Not applicable;①Clinical efficiency;②Incidence of adverse reactions; ③NIHSS scores; ④ADL scores; ⑤MCBF; ⑥MBFV; ⑦cerebral edema volume; ⑧Hcy concentration; ⑨SP concentration.

### 3.3 Literature quality evaluation

**FIGURE 2:**
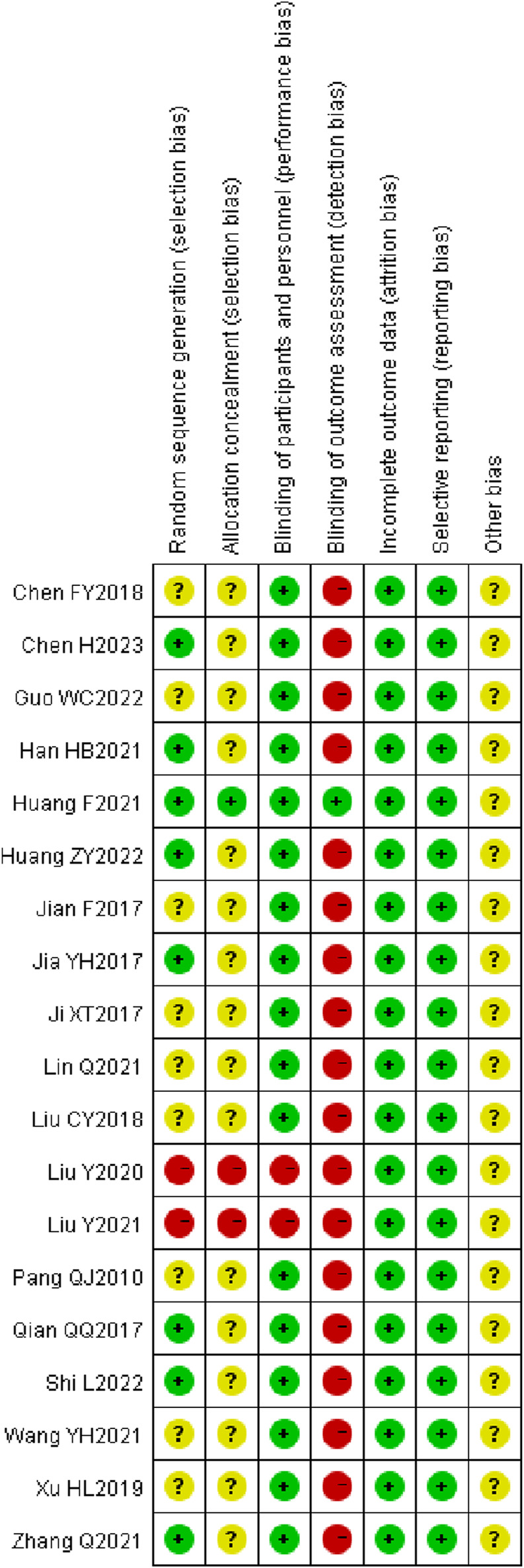
Risk of bias plot of included literature.

**FIGURE 3:**
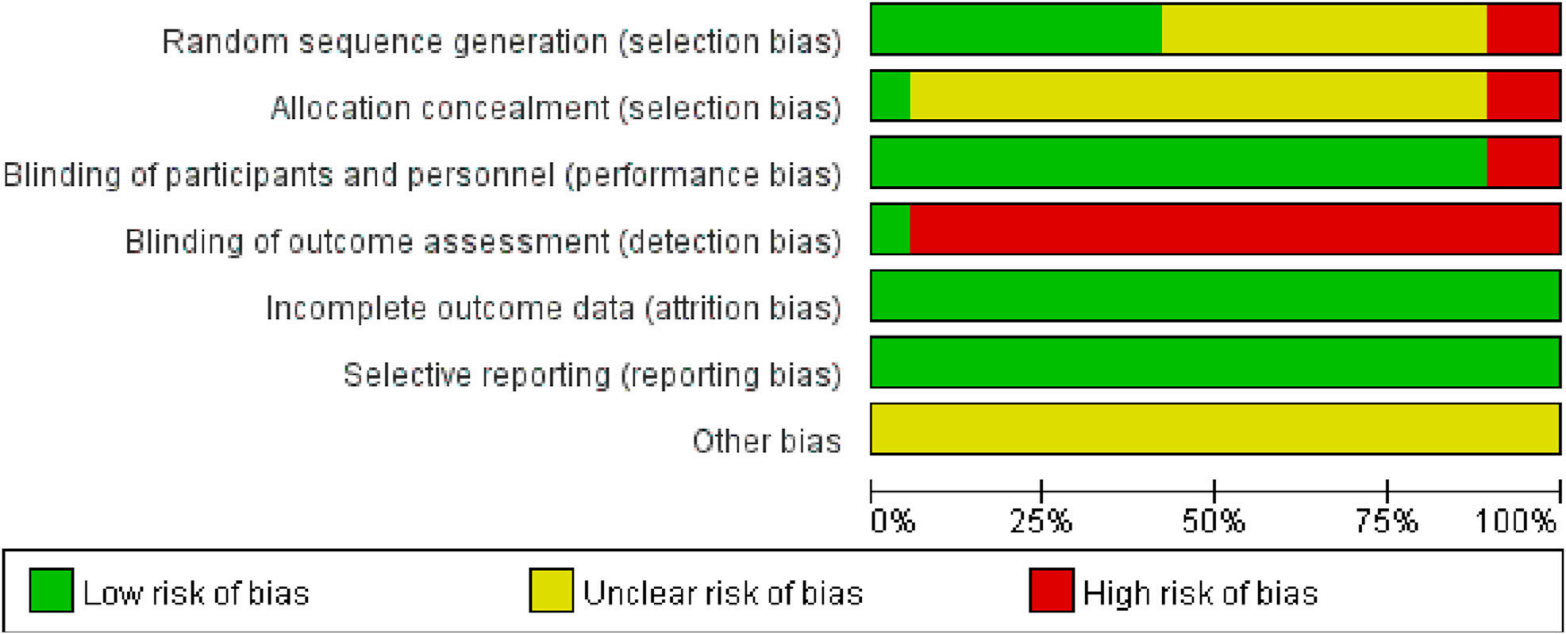
Summary of risk of bias in the included literature.

A total of seven studies (Jia et al., 2017; Qian et al., 2017; Han et al., 2021; Qian, 2021; Huang et al., 2022; Shi and Chen, 2022; Chen et al., 2023) employed the random number table method for participant allocation. Nine studies (Wang Y. et al., 2021a; Liu et al., 2018; Pang et al., 2010; hongli, 2019; Chen et al., 2018; Lin, 2021; Guo and Liao, 2022; fang, 2017; Ji and Zhou, 2017) only specified the use of “random” without further detail. One study (yan et al., 2021) allocated participants according to different treatment groups. Another study (fang and yixin, 2021) used a lottery system for grouping, and one study (yang et al., 2020) divided participants based on odd and even numbers. None of the studies exhibited evidence of selective reporting. One study (Liu et al., 2018) provided detailed information on patient dropouts and missed follow-ups, including the primary reasons for these occurrences. These aspects are illustrated in Figure 2, 3.

### 3.4 Analysis of outcomes

#### 3.4.1 Clinical efficiency

**FIGURE 4:**
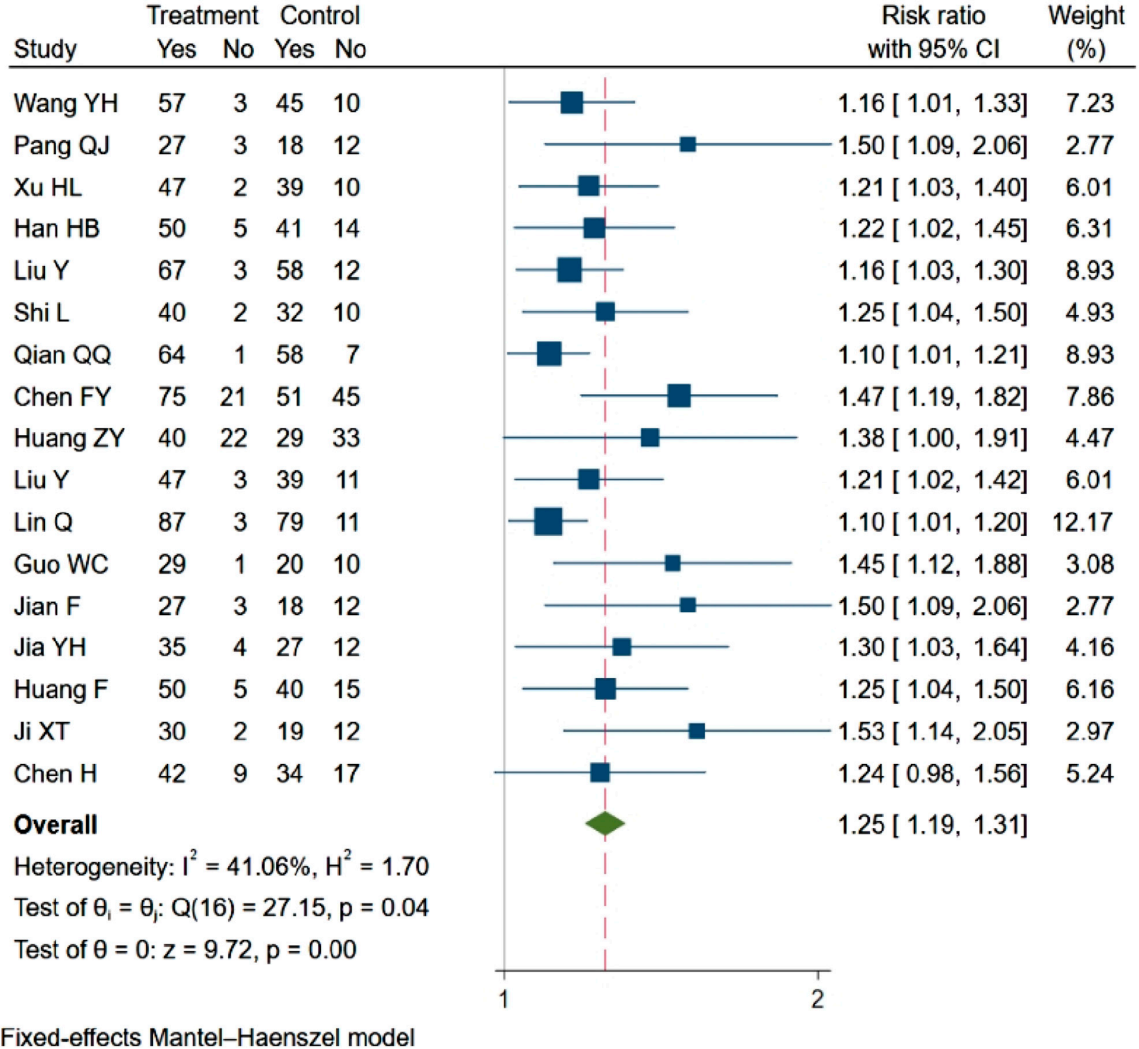
Meta-analysisof clinical efficiency.

Out of the 19 studies included, 17 (Wang Y. et al., 2021a; Pang et al., 2010; hongli, 2019; Han et al., 2021; yan et al., 2021; Shi and Chen, 2022; Qian et al., 2017; Chen et al., 2018; Huang et al., 2022; yang et al., 2020; Lin, 2021; Guo and Liao, 2022; fang, 2017; Jia et al., 2017; fang and yixin, 2021; Ji and Zhou, 2017; Chen et al., 2023) evaluated clinical response rates, showing a statistically significant difference between the two groups. In compliance with the criteria for meta-analysis inclusion, these 17 studies (n = 1808) were analyzed. The analysis revealed that NBP enhanced the clinical efficacy rate (RR = 1.25, 95% CI: 1.19–1.31; *p* = 0.00; Q (13) = 10; *p* = 0.04; I^2^ = 41.6%). The results are presented in Figure 4.

#### 3.4.2 Incidence of adverse reactions

**FIGURE 5:**
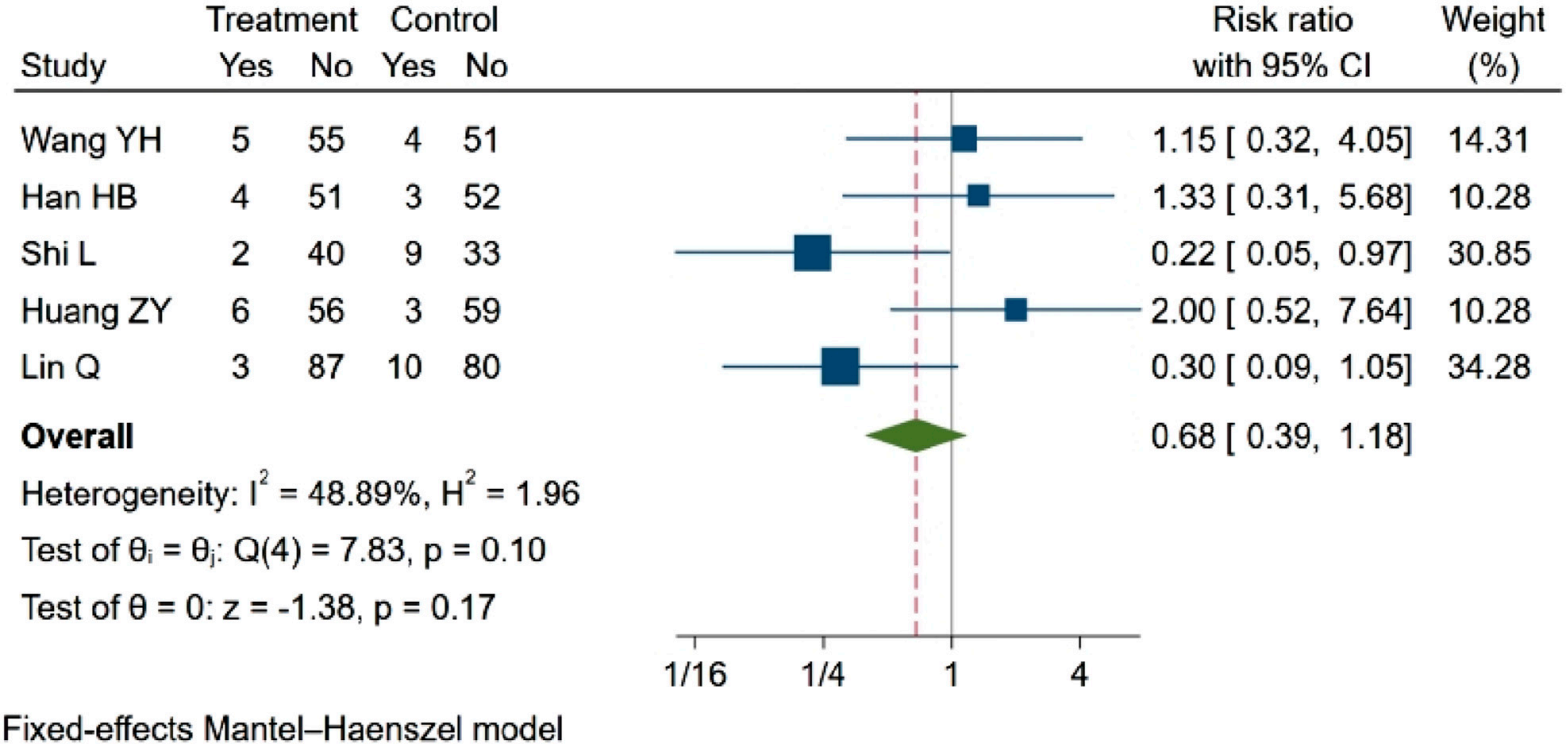
Meta-analysis of incidence of adverse reactions.

Five studies (Wang Y. et al., 2021a; Han et al., 2021; Lin, 2021; Huang et al., 2022; Shi and Chen, 2022) investigated the incidence of adverse reactions, such as nausea and vomiting. Two of these studies (Lin, 2021; Shi and Chen, 2022) reported statistical differences in comparisons before and after treatment between groups, suggesting that NBP may reduce the incidence of adverse reactions. However, three other studies (Wang Y. et al., 2021a; Han et al., 2021; Huang et al., 2022) found that NBP did not significantly decrease adverse reactions. In the meta-analysis, which included five eligible studies with a total of 613 participants, the results indicated no statistically significant difference in adverse outcomes (RR = 0.68, 95% CI: 0.39–1.18; *p* = 0.17; Q (4) = 7.83; *p* = 0.10, I^2^ = 48.89). These findings are illustrated in Figure 5.

#### 3.4.3 NIHSS score

**FIGURE 6:**
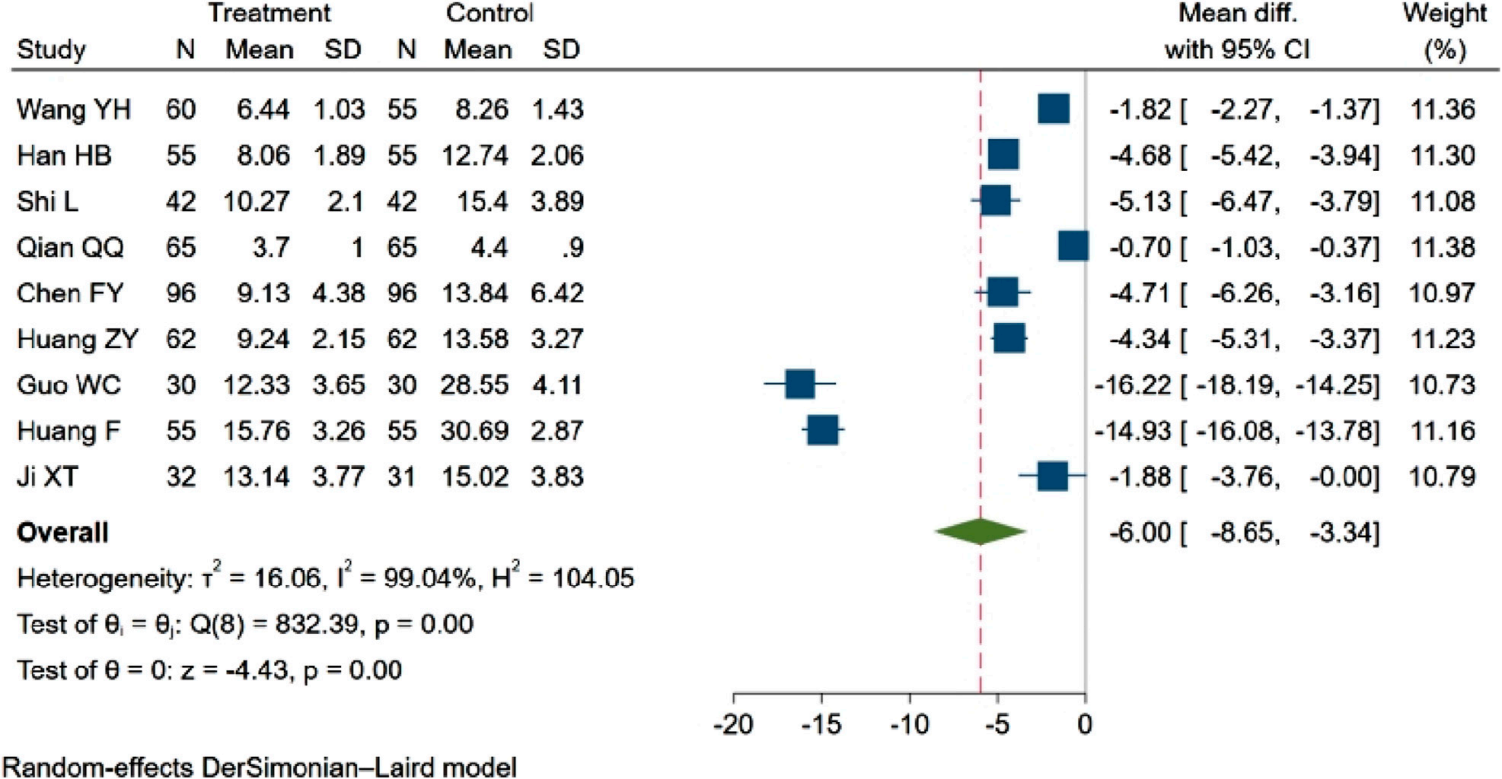
Meta-analysis of NIHSS score.

Nine studies (Wang Y. et al., 2021a; Han et al., 2021; Shi and Chen, 2022; Qian et al., 2017; Chen et al., 2018; Huang et al., 2022; Guo and Liao, 2022; fang and yixin, 2021; Ji and Zhou, 2017), with a total of 988 participants evaluated the NIHSS score. Among these, one study (Guo and Liao, 2022) observed a decreasing trend in the NIHSS score (*p* < 0.05). The remaining eight studies (Wang Y. et al., 2021a; Han et al., 2021; Shi and Chen, 2022; Qian et al., 2017; Chen et al., 2018; Huang et al., 2022; fang and yixin, 2021; Ji and Zhou, 2017) reported statistically significant differences between groups (*p* < 0.01). The aggregated results indicated that NBP contributed to a reduction in the NIHSS score (MD = −6.00, 95% CI: −8.65 to −3.34; *p* = 0.00; Q (8) = 832.39; *p* = 0.00; I^2^ = 99.04%). These findings are depicted in Figure 6.

#### 3.4.4 ADL score

**FIGURE 7:**
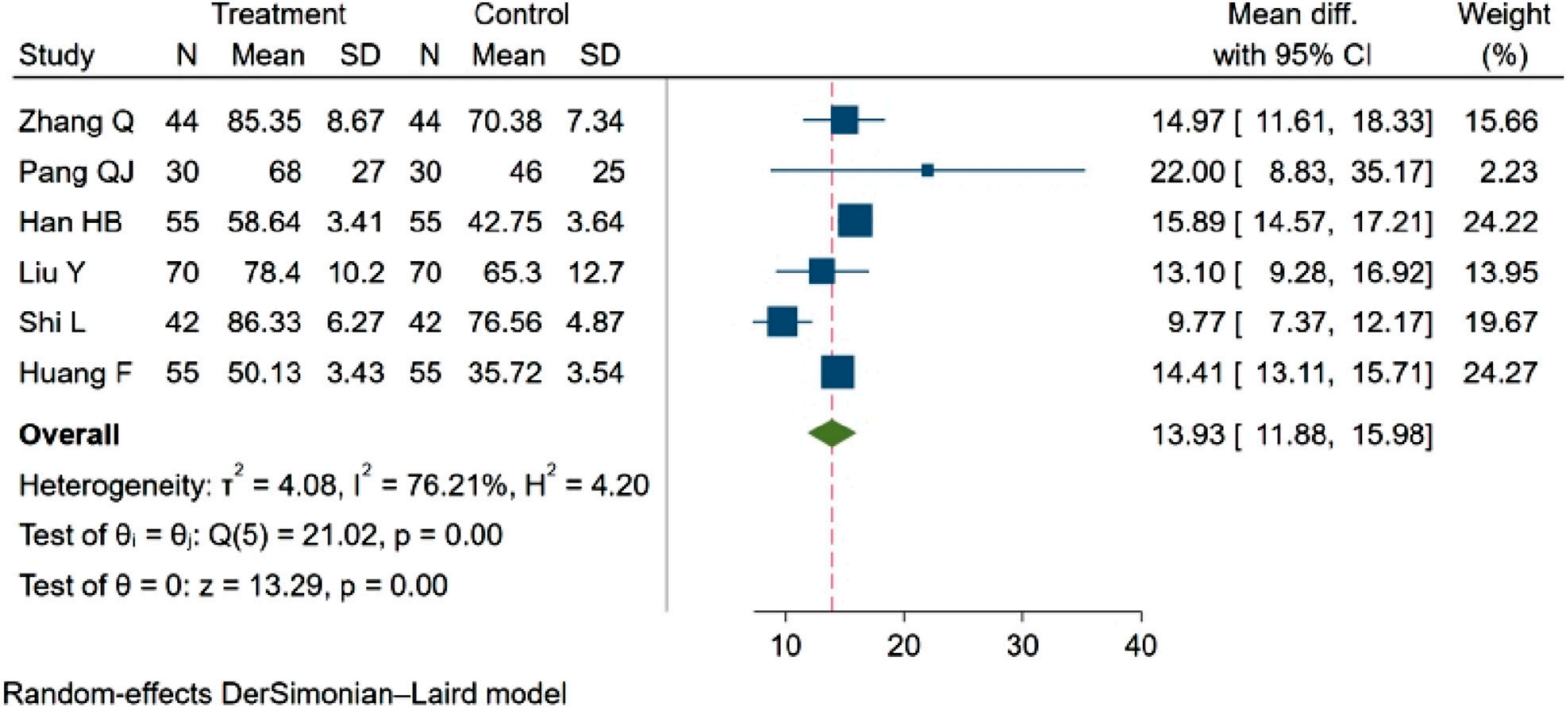
Meta-analysis of ADL score.

Six studies (Qian, 2021; Pang et al., 2010; Han et al., 2021; yan et al., 2021; Shi and Chen, 2022; fang and yixin, 2021), with a total of 592 participants evaluated the Activities of Daily Living (ADL) score. Among these, three studies (Qian, 2021; Shi and Chen, 2022; fang and yixin, 2021; Zhang et al. 2021; Shi and Chen 2022; Huang et al., 2021) indicated an increasing trend in the ADL score (*p* < 0.05). Three studies (Pang et al., 2010; Han et al., 2021; yan et al., 2021) demonstrated statistically significant differences in the ADL score between groups (*p* < 0.01). The aggregated results revealed that NBP positively influenced the ADL score, MD = 13.93, 95% CI: 11.88–15.98 (*p* = 0.00), Q (5) = 21.02 (*p* = 0.00), I^2^ = 76.21%, as depicted in Figure 7.

#### 3.4.5 Hemodynamic indicators

##### 3.4.5.1 Mean cerebral blood flow (MCBF)

**FIGURE 8:**
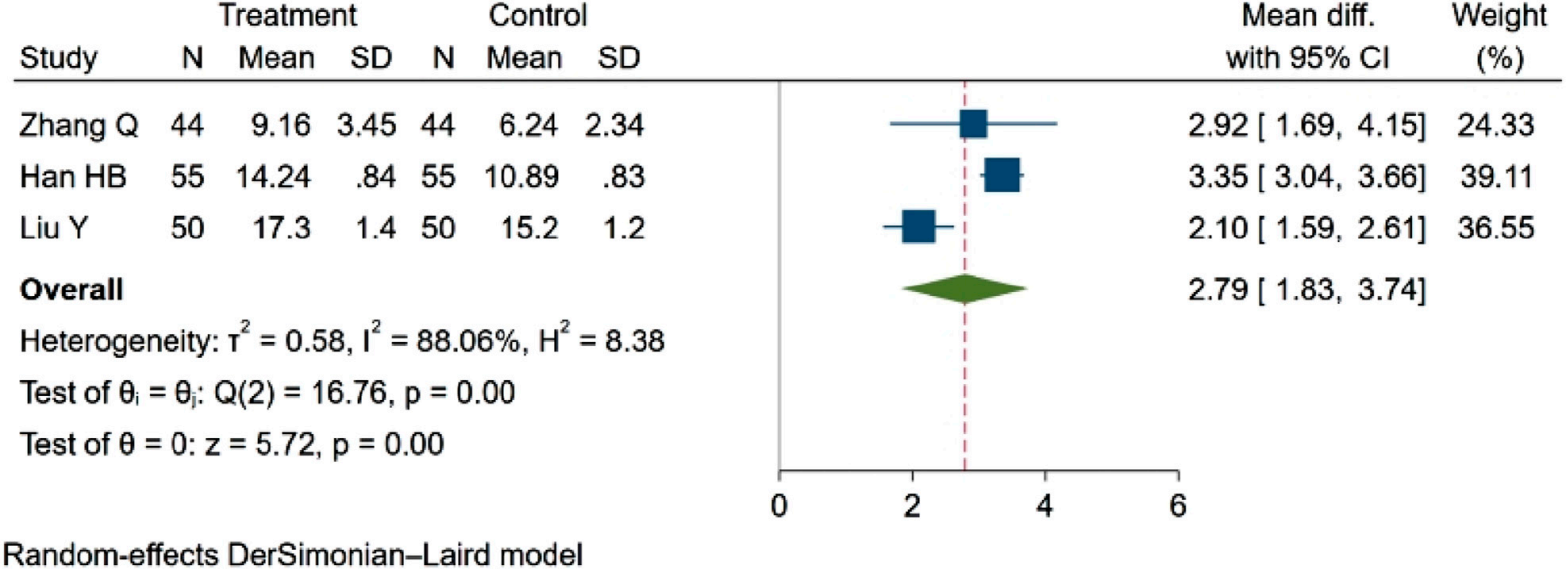
Meta-analysis results of the MCBF.

Three studies (Qian, 2021; Han et al., 2021; yang et al., 2020), comprising a total of 298 participants, assessed MCBF. The findings revealed a statistically significant difference in blood flow between groups. Specifically, NBP was associated with an increase in MCBF. MD = 2.79, 95% CI:1.83–3.74 (*p* = 0.00). Q (2) = 16.76 (*p* = 0.00), I^2^ = 88.06%, as depicted in Figure 8.

##### 3.4.5.2 Mean cerebral blood flow velocity (MBFV)

**FIGURE 9:**
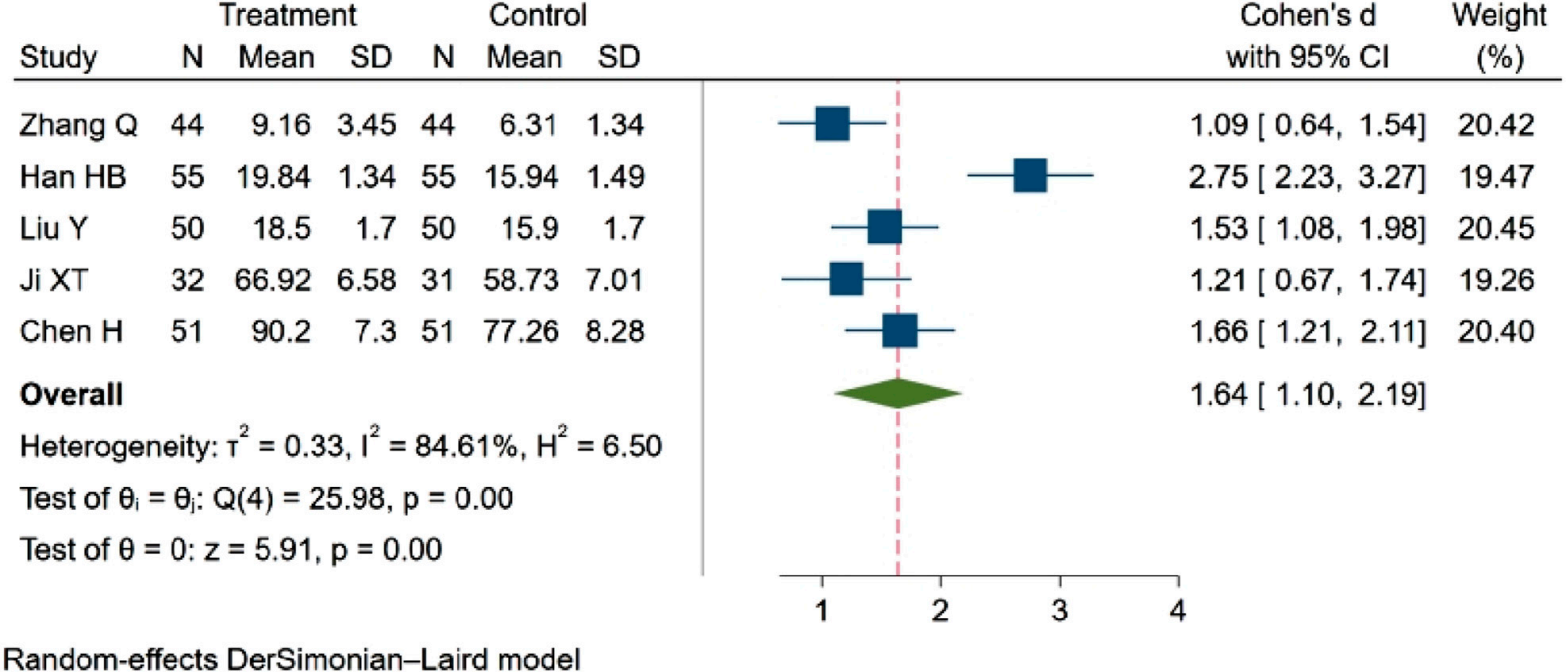
Meta-analysis of the MBFV.

Five studies (Qian, 2021; Han et al., 2021; yang et al., 2020; Ji and Zhou, 2017; Chen et al., 2023), involving a total of 463 participants, examined the MBFV. The analysis indicated a statistically significant difference in MBFV between groups. Specifically, NBP was found to increase the MBFV. SMD = 1.64, 95% CI:1.10–2.19 (*p* = 0.00). Q (4) = 25.98 (*p* = 0.00), I^2^ = 84.61%, as illustrated in Figure 9.

#### 3.4.6 Cerebral edema volume

**FIGURE 10:**
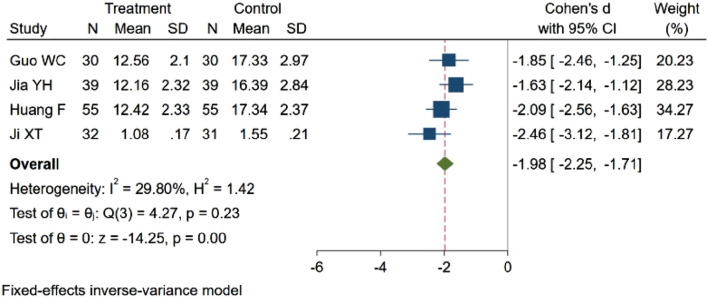
Meta-analysisof cerebral edema volume.

Four studies (Guo and Liao, 2022; Jia et al., 2017; fang and yixin, 2021; Ji and Zhou, 2017), encompassing 311 participants, evaluated the volume of cerebral edema. Among these, two studies (Jia et al., 2017; Guo and Liao, 2022) indicated a decreasing trend in cerebral edema volume (*p* < 0.05). In contrast, two studies (fang and yixin, 2021; Ji and Zhou, 2017) demonstrated statistically significant differences in cerebral edema volume between groups (*p* < 0.01). The cumulative results suggest that NBP may reduce cerebral edema volume in patients with cerebral hemorrhage. SMD = −1.98, 95% CI: −2.25 ∼ −1.71 (*p* = 0.00), Q (3) = 4.27 (*p* = 0.23), I^2^ = 29.80%, Figure 10.

#### 3.4.7 Hcy concentration

**FIGURE 11:**
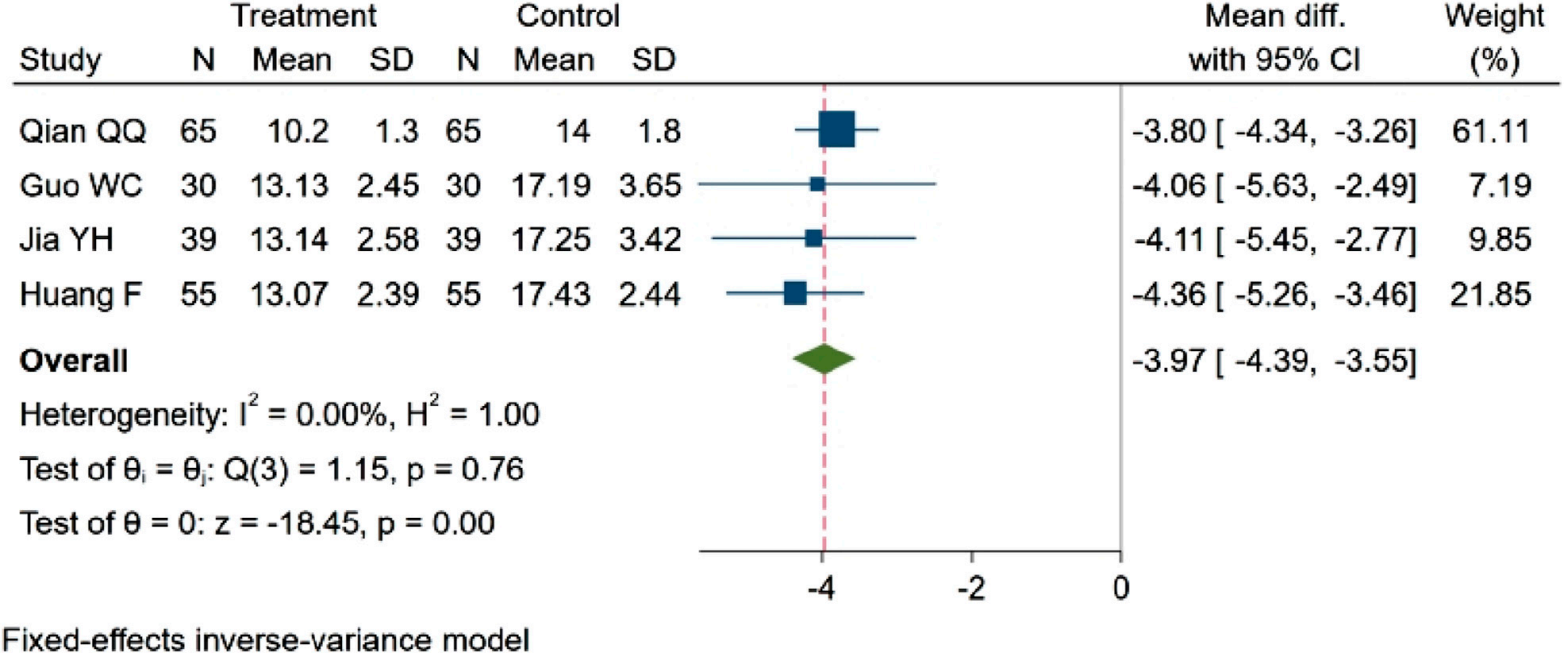
Meta-analysis of Hcy concentration.

Four studies (Qian et al., 2017; Guo and Liao, 2022; Jia et al., 2017; fang and yixin, 2021), encompassing a total of 378 participants, evaluated homocysteine (Hcy) concentration. Among these, two studies (Jia et al., 2017; Guo and Liao, 2022) observed a decreasing trend in Hcy concentration (*p* < 0.05). Two studies (Qian et al., 2017; fang and yixin, 2021) demonstrated statistically significant differences in Hcy concentration between groups (*p* < 0.01). The consolidated results indicate that NBP effectively reduced the Hcy concentration. MD = −3.97, 95% CI: −4.39 ∼ −3.55 (*p* = 0.00). Q (3) = 1.15 (*p* = 0.76), I^2^ = 0.00%, as illustrated in Figure 11.

#### 3.4.8 SP concentration

**FIGURE 12:**
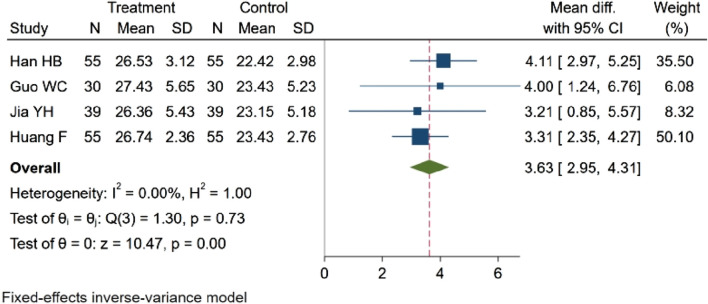
Meta-analysis of SP concentration.

Four studies (Han et al., 2021; Guo and Liao, 2022; Jia et al., 2017; fang and yixin, 2021), encompassing a total of 358 participants, evaluated SP concentration. Among these, two studies (Jia et al., 2017; Guo and Liao, 2022) reported a rising trend in SP concentration, and there is a statistical difference (*p* < 0.05). Otherwise, two studies (Han et al., 2021; fang and yixin, 2021) demonstrated statistically significant differences in SP concentration between groups (*p* < 0.01). The aggregated data reveal that NBP effectively increased SP concentration. MD = 3.63, 95% CI: 2.95–4.31 (*p* = 0.00). Q (3) = 1.30 (*p* = 0.73), I^2^ = 0.00%, as depicted in Figure 12.

#### 3.4.9 Sensitivity analysis (NIHSS, ADL score, hemodynamic indexes)

**FIGURE 13:**
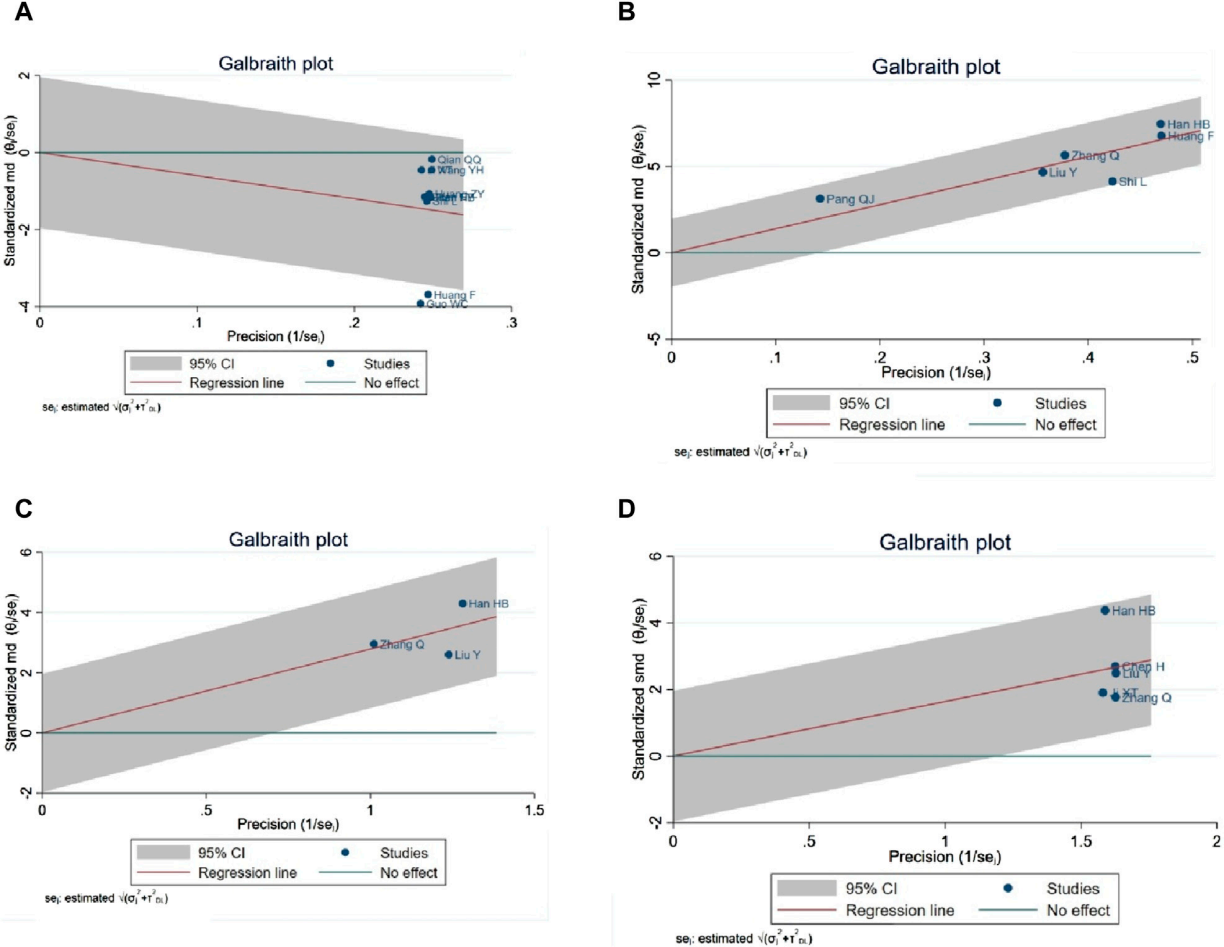
**(A)** NIHSS score, **(B)** ADL score, **(C)** MCBF, **(D)** MBFV.

A Galbraith map was utilized for sensitivity analysis to identify the sources of high heterogeneity in the NIHSS score. This analysis revealed that two studies (Guo and Liao, 2022; fang and yixin, 2021) fell outside the 95% CI, suggesting they might be anomalies contributing to the heterogeneity, as depicted in Figure 13A. Excluding two studies (Guo and Liao, 2022; fang and yixin, 2021) from the analysis, the NIHSS score remained statistically significant (SMD = −1.29, 95% CI -1.72 to −0.85; *p* < 0.0001), and heterogeneity was notably reduced (*p* = 0.000; I^2^ = 88%). In the ADL score heterogeneity analysis, the study (Shi and Chen, 2022) was identified as not within the 95% CI, potentially indicating a source of heterogeneity, as shown in Figure 13B. Removal of study (Shi and Chen, 2022) maintained the statistical significance of the ADL score (MD = 15.03, 95% CI 14.02 to 16.04; *p* < 0.0001), with a significant reduction in heterogeneity (*p* = 0.34; I^2^ = 12%). Sensitivity analysis of mean blood flow, as illustrated in Figure 13C, did not reveal specific sources of heterogeneity. Regarding mean blood flow velocity, sensitivity analysis indicated that the statistical significance persisted even after the exclusion of study (Han et al., 2021) (SMD = 1.37, 95% CI 1.10 to 1.64; *p* < 0.0001), with a substantial reduction in heterogeneity (*p* = 0.27, I^2^ = 23%), as shown in Figure 13D.

### 3.5 Meta-regression analysis

The results of meta-analysis of clinical efficacy showed that the effect size was not significantly correlated with age, gender, and drug dosage form (*p* > 0.05).

### 3.6 Publication bias

**FIGURE 14:**
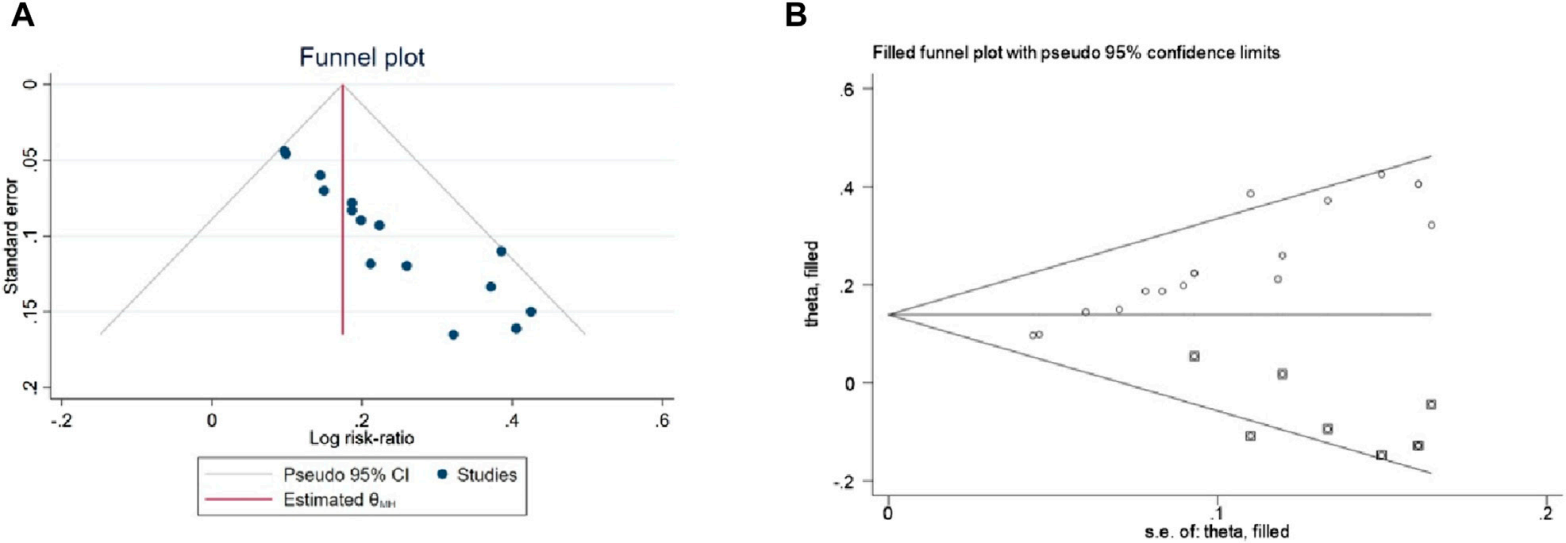
**(A)** Funnel plot of clinical efficacy, **(B)** Trim and filling figure of meta-analysis of clinical efficiency.

Given the requirement of a minimum of 10 original studies for funnel plot analysis, this approach was applied exclusively to clinical efficacy rates. Subsequently, an Egger test was conducted, revealing a statistically significant difference (*p* < 0.01), indicating publication bias in clinical remission rates. To address this, a “trim and filling” method was employed to estimate the adjusted combined effect size for publication bias. The adjusted combined effect size was found to remain statistically significant (*p* < 0.01), aligning with the unadjusted findings. In conclusion, the publication bias in clinical efficiency rates was minimal, and the results were robust, as evidenced in Figures 14A, B.

## 4 Discussion

### 4.1 Findings analysis

To our knowledge, this is the inaugural systematic review and meta-analysis investigating the clinical efficacy and outcomes of NBP in cerebral hemorrhage patients. The findings indicate that NBP markedly enhances clinical efficacy, cerebral blood flow, and velocity, facilitates neurological function recovery, ameliorates symptoms of cerebral edema, and attenuates the inflammatory response. Furthermore, the use of NBP in treating cerebral hemorrhage was not associated with significant adverse reactions. Consequently, NBP can be considered a viable therapeutic option for cerebral hemorrhage treatment.

This study reveals that NBP enhances clinical efficacy in patients with cerebral hemorrhage, contributing to reductions in both the NIHSS score and the ADL score. Furthermore, NBP was found to promote neurological recovery and elicit a greater clinical response than conventional treatment. These findings align with the experimental results reported by Zhao et al. (Zhao et al., 2017), which indicated that continuous NBP administration for 21 days post-traumatic brain injury (TBI) significantly improved sensorimotor recovery in adult male mice. Our meta-analysis corroborates the ability of NBP to notably improve patient living capabilities. Correspondingly, Lv et al. (Lv et al., 2021) reported that a combination of NBP with modified mandatory exercise therapy markedly enhanced daily living abilities in patients with cerebral hemorrhage, echoing our observations.

NBP has been effective in alleviating symptoms of cerebral hemorrhage by reducing Hcy concentrations. Multiple studies have identified Hcy as a biomarker for predicting cerebral hemorrhage, categorizing it as an independent predictor (Zhang et al., 2022). Consequently, Hcy was chosen as one of the main indicators in this study. Xu et al. reported that Hcy levels can be instrumental in assessing the progression and prognosis of cerebral hemorrhage. Their research indicated that higher serum Hcy concentrations in patients are associated with larger cerebral hemorrhage volumes, which is crucial for timely assessment of the condition’s progression and prognosis (Xu et al., 2022). In this studies, a positive correlation was found between Hcy concentration and the severity of cerebral hemorrhage. Our findings confirm that NBP significantly reduces Hcy concentrations.

SP, a neuropeptide of the tachykinin family, is widely distributed in the central and peripheral nervous systems, respiratory system, and intestinal tract. It exhibits proinflammatory effects upon binding to its receptors. In cerebral hemorrhage patients, SP may play a key role in hematoma enlargement, perihematoma edema, and neuroinflammatory responses (Lorente et al., 2020; Wang et al., 2020). Animal model studies have shown that inflammation can lead to neuronal death, blood-brain barrier disruption, and neurological deficits post-ICH in rats (Zhu et al., 2018; Chen S. et al., 2022b). Our results indicated that NBP significantly increases SP concentration. Potentially by blocking SP’s binding to its receptor, thus reducing inflammation and edema in brain tissue. The rise in peripheral SP level might be a feedback regulation after the inhibition of the SP receptor, consistent with the conclusions of Wang et al. (Wang haibo et al., 2021b). However, the specific mechanism requires further exploration.

Perihematoma edema is a principal contributor to the high morbidity and mortality associated with intracranial hemorrhage. The edema volume often surpasses that of the original hematoma, potentially increasing intracranial pressure or leading to hydrocephalus, thereby causing neurological deterioration and even death (Mehdiratta et al., 2008). Persistent or expanding edema can further impede cerebral blood supply, disrupt the intracellular environment of nerve cells, and exacerbate nerve cell damage (Gong et al., 2015; Wang et al., 2016). Animal experimental studies have demonstrated that NBP significantly enhances neurological function and mitigates symptoms of cerebral edema in rats experiencing cerebral hemorrhage (Huang et al., 2021). These findings are consistent with our own observations.

The monitoring and regulation of hemodynamic parameters are critical in cerebral hemorrhage management (Jurgen et al., 2021). Cerebral vasospasm, commonly observed in subarachnoid hemorrhage patients, is intricately linked to the reduction in blood flow velocity and volume, potentially impairing the body’s capacity for autoregulation of these parameters. Experimental studies have indicated that alterations in cerebral blood flow (CBF) correlate with the capacity for oxygen delivery. Cerebral hypoperfusion and inadequate oxygen delivery can result in brain tissue injury (Prunell et al., 2004; Muench et al., 2007). Lennihan L et al. demonstrated that increasing CBF and enhancing microcirculation can mitigate brain tissue damage (Lennihan et al., 2000; Qiu et al., 2019). The meta-analysis results of this study suggest that NBP may enhance cerebral blood flow and velocity, which could be a key mechanism in restoring cerebral nerve function deficits. However, due to limited sample sizes, extensive evidence is still required for confirmation. Our meta-analysis observed that the improvement in cerebral hemorrhage adverse effects by NBP was marginally lower than that reported in the retrospective study by Lv et al. (Lv et al., 2021). In this study, the improvement in adverse effects was less pronounced with NBP, but did not exacerbate the adverse effects.

### 4.2 Limitations

This study acknowledges five limitations in the evidence presented. Firstly, the majority of the included studies originated from China, with all cases involving Chinese patients, introducing potential regional publication bias. Further international clinical trials are warranted. Secondly, the assessment of evidence quality revealed that only seven studies in the meta-analysis reported random sequence generation, and none implemented blinding for subjects and clinicians or reported on it. This aspect necessitates optimization in clinical trial design and may significantly impact the quality of the literature. Thirdly, due to publication bias in clinical response rates, the “trim and filling” method was employed to estimate the adjusted combined effect size. However, this approach, based on the principle of symmetry and the inclusion of hypothetical small-sample studies, warrants cautious interpretation of clinical response rate results. Importantly, the interventions in the control groups of the included studies were all conventional Western medical treatments viewed as placebo therapy was not involved in the control groups and most of the studies did not have a follow-up period, which may expensively result in a waste of research Finally, while efforts were made to encompass a comprehensive range of studies, the exclusion of grey literature might have led to the omission of crucial literature.

This review process is subject to two main limitations. Firstly, the scope of our study predominantly focused on evaluating the clinical efficacy, neurological function, quality of life, and blood indices of NBP in cerebral hemorrhage patients. It did not extensively delve into the occurrence of adverse events linked to the prognosis of cerebral hemorrhage. This limitation stems from the absence of clinical trials concerning NBP that investigate prognosis-related adverse outcomes. Secondly, the studies incorporated in our analysis were characterized by small sample sizes. Future research should encompass a substantial number of multi-center, randomized, double-blind controlled trials with larger samples to substantiate the efficacy of NBP and elucidate its specific biomolecular mechanisms.

## 5 Conclusion

In conclusion, our study demonstrates that NBP effectively enhances clinical response rates, neurological function, and quality of life, while reducing cerebral blood flow and velocity in cerebral hemorrhage patients. Additionally, NBP exhibits minimal side effects and may ameliorate brain injury and clinical symptoms by diminishing inflammatory responses and Hcy concentrations. This review substantiates that timely administration of NBP can improve clinical symptoms in the cerebral hemorrhage population. The findings aim to shed light on NBP’s potential targets in treating cerebral hemorrhage and provide valuable insights for clinicians, medical institutions, and intervention strategists dealing with cerebral hemorrhage.

## Data Availability

The original contributions presented in the study are included in the article/Supplementary Material, further inquiries can be directed to the corresponding author.
